# Microbial Community Response to Various Types of Exogenous Organic Matter Applied to Soil

**DOI:** 10.3390/ijms241914559

**Published:** 2023-09-26

**Authors:** Sylwia Siebielec, Anna Marzec-Grządziel, Grzegorz Siebielec, Aleksandra Ukalska-Jaruga, Monika Kozieł, Anna Gałązka, Marcin Przybyś, Piotr Sugier, Magdalena Urbaniak

**Affiliations:** 1 Department of Microbiology, Institute of Soil Science and Plant Cultivation—State Research Institute, Czartoryskich 8, 24-100 Pulawy, Poland; agrzadziel@iung.pulawy.pl (A.M.-G.); monika.koziel@iung.pulawy.pl (M.K.); agalazka@iung.pulawy.pl (A.G.); 2 Department of Soil Science Erosion and Land Protection, Institute of Soil Science and Plant Cultivation—State Research Institute, Czartoryskich 8, 24-100 Pulawy, Poland; gs@iung.pulawy.pl (G.S.); aukalska@iung.pulawy.pl (A.U.-J.); 3 Department of Plant Breeding and Biotechnology, Institute of Soil Science and Plant Cultivation—State Research Institute, Czartoryskich 8, 24-100 Pulawy, Poland; marcin.przybys@iung.pulawy.pl; 4 Department of Botany, Mycology and Ecology, Institute of Biological Sciences, Maria Curie-Sklodowska University, Akademicka 19, 20-033 Lublin, Poland; piotr.sugier@mail.umcs.pl; 5 UNESCO Chair on Ecohydrology and Applied Ecology, Faculty of Biology and Environmental Protection, University of Lodz, Banacha 12/16, 90-237 Lodz, Poland; magdalena.urbaniak@biol.uni.lodz.pl

**Keywords:** Biolog EcoPlates, bottom sediment, enzyme activity, bacterial diversity, manure, NGS, white mustard, sewage sludge

## Abstract

Recycling of solid biowaste and manure would reduce the dependence of agriculture on synthetic products. Most of the available studies on the effects of exogenous organic matter (EOM) application to soil were focused on nutrients and crop yield, with much less attention to microbiological processes in soil, especially using modern molecular methods. The aim of this study was to evaluate the effects of various types of manure, sewage sludge and bottom sediment on the biochemical activity and biodiversity of soil and plant yield in a pot experiment. The soil was treated with a range of EOM types: six types of manure (cattle, pig, goat, poultry, rabbit and horse manure; two bottom sediments (from urban and rural systems); and two types of municipal sewage sludge. All EOMs stimulated dehydrogenases activity at a rate of 20 t ha^−1^. Alkaline phosphatase was mostly stimulated by poultry manure and one of the sludges. In general, the two-fold greater rate of EOMs did not further accelerate the soil enzymes. The functional diversity of the soil microbiome was stimulated the most by cattle and goat manure. EOMs produce a shift in distribution of the most abundant bacterial phyla and additionally introduce exogenous bacterial genera to soil. Poultry and horse manure introduced the greatest number of new genera that were able to survive the strong competition in soil. EOMs differentiated plant growth in our study, which was correlated to the rate of nitrate release to soil. The detailed impacts of particular amendments were EOM-specific, but in general, no harm for microbial parameters was observed for manure and sludge application, regardless of their type. There was also no proof that the PAH and pesticide contents measured in manure or sludge had any effect on microbial activity and diversity.

## 1. Introduction

Soil organic matter (SOM) is one of the key soil components affecting soil health, productivity and ecosystem services. SOM level influences physical, chemical and biological properties of soil. It improves soil structure, resistance to erosion and soil biodiversity [1,2,3]. On the other hand, decline of SOM has been defined as one of major threats to soil and challenges to be addressed [4]. SOM mineralization in soil and its loss can be counteracted by agricultural practices that facilitate better SOM stability in soil or greater input of plant residues to soil profile. Another strategy to increase the accumulation of organic matter or prevent it from declining in agricultural soils is to deliver additional OM supplies in the form of exogenous organic materials [1,5]. A transition towards a circular economy and the expected reduction of synthetic nutrient use in agriculture have increased the importance of recycling carbon and nutrients from waste for agronomic purposes. Importantly, low soil productivity and the effects of climate change, combined with the chemicalization of agriculture, require climate-friendly farming and nutrient recovery to improve food security and increase the resilience or adaptability of agricultural systems [6].

Various types of exogenous organic matter (EOM) additives are used to increase SOM in the soil environment, the classical form of which is manure. However, due to the shortage of manure in many regions resulting from the regionalization of animal production, other alternative and commonly available exogenous sources of organic matter are taken into account. These might include sewage sludge, bottom sediments, food sector waste, composted industrial and municipal waste, biogas plant digestate, and animal bone meal [7,8,9,10,11,12]. EOM derived from various materials and processes is characterized by diverse properties such as decomposition rate, availability of nutrients and dynamics of their release, sorption properties, and contaminant contents. Therefore, the impact of these materials on the soil environment varies substantially [12,13,14].

The literature indicates that currently 1.3 billion tons of municipal solid waste are produced in the world annually, and it is estimated that by 2025 this number may increase to 2.2 billion tons [15]. Recycling of solid waste and animal excrement and their potential use as fertilizers would reduce the dependence on mineral fertilization, as assumed in EU strategies [6]. Emerging types and increasing amounts of waste make it necessary to develop methods and strategies for their disposal, including use in agriculture, while maintaining environmental safety. The available data show that, on average, municipal sludge produced in Poland contains 2.6% nitrogen (N) and 1.83% phosphorus (P) in its dry matter. Taking into account the volume of produced sludge, this type of EOM constitutes a substantial nutrient resource [16]. A precondition for the widespread use of sludge in agriculture must be, however, high nutrient use efficiency and absence of negative effects, such as the transfer of pollutants to crops or groundwater.

Bottom sediments are in general removed only in the case of necessity of dredging of lakes or rivers. The problem of safe sediment management is therefore still underestimated. Research on bottom sediment use as soil amendment is quite scarce, and there are no specific regulations dedicated to this issue. Bottom sediments might contain substantial amounts of carbon; however, this parameter can vary between sediments. There might be justifications for sediment recycling, but the risk might be related to the perceived presence of pollutants and the unknown consequences of their use for the biology of the soil [17,18]. It should be emphasized that the safe use of exogenous organic matter on arable land requires strict control of pollutants, which include potentially toxic trace elements (PTTE) and various organic compounds.

Most of the available studies on the effects of EOM application to soil generally con-cerned the utilization of nutrients (nitrogen, phosphorus) and the effect on yield (productive soil function). Research shows that the use of EOM as an additive to soil contributes to an increase in soil fertility and microbial activity, as well as resistance of the microbiome to drought, and this effect is modified by soil conditions and EOM type [19,20,21]. However, the literature on comparing the impacts of various EOMs on soil microbiome, its metabolic and enzymatic activity, and its genetic structure is relatively scarce. Undoubtedly, soil microorganisms take an active part in the soil functioning, determining both the direction and nature of biochemical changes. According to the literature, as many as 80% of all soil processes are closely related to the activity of microorganisms [22,23,24,25]. The assessment of microbiological properties is an important part of the monitoring of changes in the soil environment and may also constitute a key element of the soil health. Therefore, it is very important to assess how the addition of a given type of exogenous organic matter affects the diversity of microorganisms and the activity of specific microbially driven processes in soil. Past results show that the addition of EOM to the soil may alter microbiological properties of the soil, but the degree of their modification might depend on the type of EOM applied [26,27,28,29,30]. Most of these studies on EOM impact on microbiological properties of soil were, however, focused on manure effects [31,32,33,34,35,36] or did not compare the effects of a wider range of organic materials. 

Taking the existing knowledge into account, our hypothesis assumed that the effects of organic fertilization on the microbiological properties of the soil may be strongly dependent on the chemical composition and type of EOM. Therefore, the aim of this study was to compare the effects of various types of manure, sewage sludge and bottom sediment and their rates on the biochemical activity, as well as soil bacterial diversity and plant yield in a pot experiment.

## 2. Results and Discussion

### 2.1. Chemical Properties of EOM

The basic properties of the tested organic materials are presented in Table 1. Cattle manure (41.8%), goat manure (38.5%) and rabbit manure (37.1%) were the richest in carbon. The highest contents of nitrogen per unit of dry matter were found in sewage sludge I and sewage sludge II (3.55% and 5.24%), as well as rabbit manure (3.25%). The greatest content of phosphorus was recorded for sewage sludge I (2.74%), followed by pig manure (1.62%). Most of the manure samples were rich in potassium (cattle, pig, goat, horse and rabbit manure). 

The mineral fraction predominated in the bottom sediments, so they contained much less carbon and nitrogen than the other materials. Table 1 shows the contents of the basic trace elements. Cadmium (1.06 mg kg^−1^) and zinc (1309 mg kg^−1^) were the highest in sewage sludge II, but these contents did not exceed the permissible values for the use of municipal sewage sludge in agriculture, in accordance with the Regulation of the Minister of the Environment of 6 February 2015 [37].

In the analyzed EOM samples, two pesticide compounds were detected in all cases: Chlorpyrifos-methyl and DDD-p,p’ (Table S1, Supplementary Materials). As reported in the literature, DDT residues and its metabolites (p, p’-DDE, p, p’-DDD) in soil constitute an environmental problem worldwide. They were widely used in agriculture from the 1930s to the early 1970s [38]. Chlorpyrifos-methy is classified as an organophosphorus substance, a non-carcinogenic substance mainly used to combat insects, which inhibits the activity of acetylcholinesterase [39]. Interestingly, a wide range of pesticides was detected in sewage sludge II. Contents of PAHs in EOMs are presented in Table S2. Poultry manure contained the greatest amount of most of the PAHs. Only naphthalene, acenaphthylene and fluorene were the highest in sewage sludge I, cattle manure and sewage sludge II, respectively.

### 2.2. EOM Effects on Microbiological Soil Parameters

#### 2.2.1. Enzymatic Activity

The content of organic matter in soil is an important feature that determines the proper functioning of the soil environment, including the dynamics of microbially driven processes. Synthetic fertilization primarily serves as delivery of nutrients needed by plants, but application of EOMs stimulates significant changes in the soil environment—e.g., cycling of nitrogen in the soil and change in the microbiological parameters, such as the activity and diversity of soil microorganisms [40,41,42]. 

The multi-way ANOVA revealed statistically significant main effects of the EOM, rate and plant presence and also EOM × rate, EOM × plant, rate × plant and EOM × rate × plant interactions on the activity of acidic phosphatase (Table 2). Similar results were obtained in the case of dehydrogenase activity; however, the exception was EOM × rate × plant interaction. The effects of EOM type, rate and plant presence were also significant in the case of alkaline phosphatase, but there were no statistically significant effects of interactions between EOM and plant or between EOM, rate and plant presence.

Since for the purposes of this paper the authors intended to discuss exclusive EOM effects in more detail, we present the enzymatic data for non-planted soil in the body of the manuscript, whereas the data collected for planted soil is fully presented in the Supplementary Materials. 

Phosphatases are responsible for the hydrolysis of organic phosphorus compounds and are indicators of the potential rate of their mineralization in the soil environment [43]. In the non-planted pots, soil amendments did not strongly affect acidic phosphatase activity. Only sewage sludge II stimulated activity of the enzyme compared to other tested EOMs, regardless of the EOM rate (Table 3). Contrarily, alkaline phosphatase activities were significantly lower in the controls and the soil treated with both bottom sediments. Alkaline phosphatase was the most active in soil amended with poultry manure and sewage sludge I. Dehydrogenases occur in all living cells of microorganisms; therefore, they are usually considered indicators of the overall microbial activities of soils [44]. All EOMs stimulated greater numbers of dehydrogenase activity at a rate of 20 t ha^−1^ (Table 3). However, the most active were soils treated with sewage sludge II, followed by horse manure and sewage sludge I. Interestingly, there were no effects of ammonium nitrate application on any of the analyzed enzymes, as compared to the fully untreated soil. A positive effect of sewage sludge on enzyme activities has been observed by other authors [45,46]; however, in general, the comparison of sludge and manure effects on soil microbial processes is insufficient. 

The two-fold greater rate of EOMs did not further stimulate the soil enzymes as a general trend (Table 3). The effect of rate was rather EOM-specific. There were some strong stimulations: goat, rabbit and horse manure further enhanced dehydrogenase activity by 74, 45 and 105%, respectively, and alkaline phosphatase was greatly stimulated by poultry manure (by 34%). This confirms the rate-driven reviving effect of poultry manure on enzymes that are critical for phosphorus cycling in soil. 

**Figure 1:**
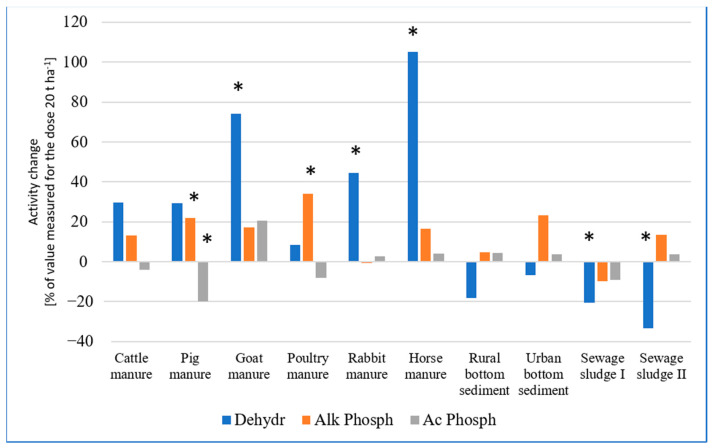
The effect of increasing EOM rate on soil enzyme activities as percent change of the activity measured for rate of 20 t ha^−1^ (* means statistically significant differences between values measured for doses 20 and 40 t ha^−1^).

In order to better visualize the effect of EOM rate on the enzymatic activity in non-planted soil, the change of activity resulting from the increase in rate is shown in Figure 1. In general, dehydrogenases and alkaline phosphatase exhibited similar trends. Raising the manure rate further increased their activities to some extent, but some of these enhancements were not significant. Opposingly, the greater dose of sewage sludge I reduced the activity of all three enzymes as compared to 20 t ha^−1^. Sewage sludge II at a greater rate caused much lower dehydrogenase activity than after adding a two-fold smaller amount of sludge but did not reduce phosphatases (Figure 1). 

In the presence of plants, the activities measured were slightly higher, which can be an effect of root secretions. But, in general, the pattern of EOM effects was somewhat similar to non-planted soils with the lowest activities measured for sediment-treated soils (Tables S3 and S4). It is worth mentioning that the statistical analysis revealed that the growth of white mustard modified the effects of EOMs on activities of acid phosphatase and dehydrogenases to some extent. Plant presence can also modify the rate effects of EOMs on all the measured enzyme activities (Table 2). 

#### 2.2.2. Microbial Functional Diversity

Reactions of soil microbial populations to environmental stresses may not be detected by individual microbiological parameters; therefore, such a complex analysis of the metabolic structure of the entire population as Biolog EcoPlates might give a picture of the changes that occur under the influence of EOM [47,48,49,50]. 

**Figure 2:**
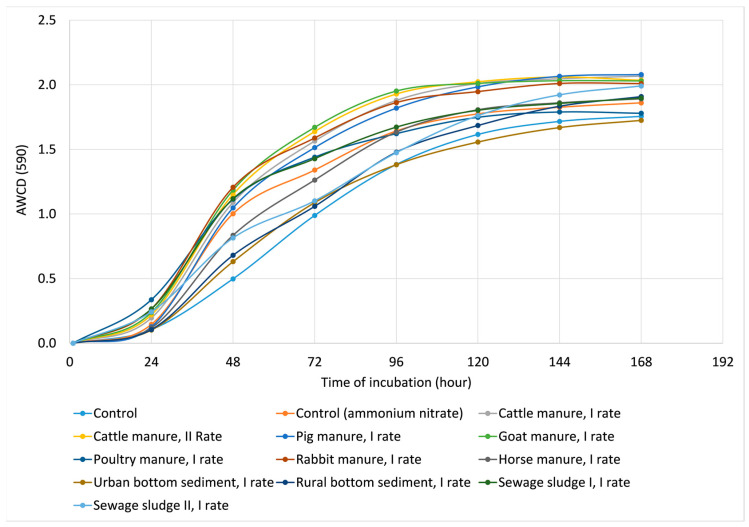
Dynamics of AWCD in soils amended with EOMs.

The dynamics of AWCD of the soil samples treated with EOMs are presented in Figure 2. The AWCD values after 24 h of incubation dynamically increased, achieving a plateau effect after 144 h. Therefore, for the detailed analysis of data from the Biolog^®^EcoPlates system, measurement after 144 h of incubation was used, since after this time, the index showed a tendency to stabilize. The highest AWCD at 144 h was recorded for soils treated with pig and cattle manure, while the lowest was in control and the soil that received urban bottom sediment.

**Figure 3:**
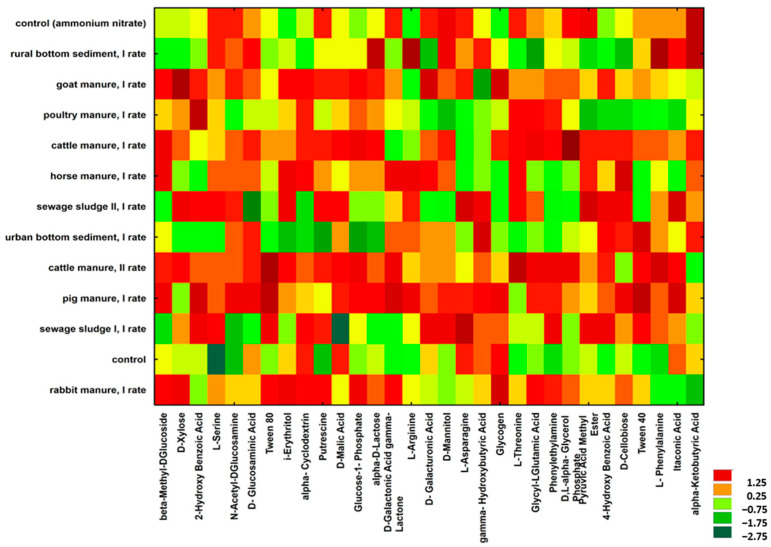
Heat map of the metabolic profile of microorganisms based on the use of various C sources using the EcoPlate method after 144 h of incubation.

The intensities of using the given carbon substrates are visualized in the form of a heat map (Figure 3). The results were calculated from the data obtained after 144 h of incubation. Visual analysis of the heatmap reveals that cattle manure and pig manure stimulated the most intensive utilization of C across all C sources. Greater doses of cattle manure even further intensified the carbon use. The application of sewage sludge to the soil caused some variability in C utilization: some compounds were decomposed intensively (L-asparagine and pyruvic acid methyl ester), other sources only to a very limited extent (D-glucosaminic acid). Visible differences were also observed in the comparison of bottom sediments with other EOMs and even between urban and rural bottom sediments. It can be also concluded that adding synthetic N fertilizers causes a shift in the functional diversity of microorganisms in soil.

The contribution of groups of C compounds in the total C utilization are presented in Table S5. It is worth mentioning that carbohydrates were most preferentially used (34.2% of total C utilization) in soil samples with the addition of urban bottom sediment, even if the intensity of use was not high. Polymers constituted a significant C source in control and in soil amended with sewage sludge I. It also seems that manures and especially bottom sediments stimulate a shift to greater activity of microorganisms utilizing less complex sources of C. 

Table 4 presents biodiversity indices calculated on the basis of the functional data obtained after a 144 h incubation of Ecoplates. The highest Shannon diversity index was found in soil with the addition of cattle manure (I rate). Compared to the untreated control, the Shannon index was significantly increased as a result of the following additions: cattle manure (both rates) and goat manure. The lowest index value was recorded for the urban bottom sediment. The highest value of the evenness index (E = 0.993) representing uniformity of C source utilization was calculated for the addition of poultry manure; however, the differences were not statistically significant between the treatments. The number of carbon substrates used (R-richness index) slightly increased after adding most of the EOMs, except for urban bottom sediment and sewage sludge II.

#### 2.2.3. Diversity of Bacteria

**Figure 4:**
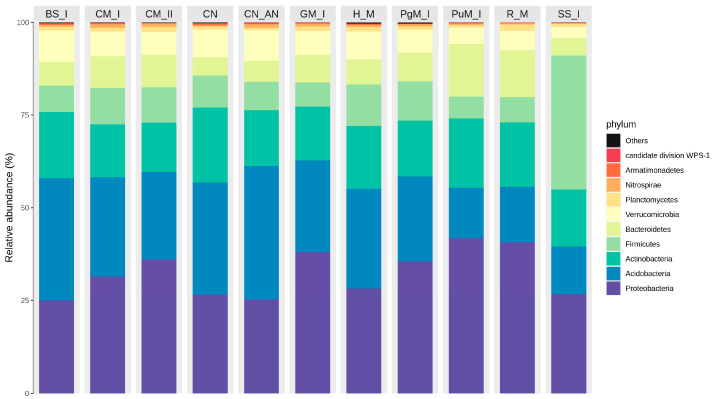
Most abundant phyla in soil after application of EOMs (bars from the left: BS—rural bottom sediment; CM—cattle manure; CN—control; CN_AN—control-AN; GM—goat manure; HM—horse manure; PgM—pig manure; PuM—Poultry manure; RM—rabbit manure; SS—sewage sludge I; I—rate 20 t ha^−1^, II—rate 40 t ha^−1^).

Bacterial communities of the soil samples were dominated by six phyla: Proteobacteria, Acidobacteria, Actinobacteria, Firmicutes, Bacteroidetes and Verrumicrobia (Figure 4). Less abundant but still well represented were also Planctomycetes and Nitrospirae. The analysis revealed that mineral N fertilizer did not strongly change phylum abundance, except for a shift between Acidobacteria and Actinobacteria, which were increased and reduced after ammonia nitrate application, respectively. Actinobacteria are considered extremely valuable in soil due to production of a number of bioactive substances (vitamins, siderophores, enzymes, pigments) with a broad spectrum of antibacterial and antiviral activities. They also metabolize organic matter, contributing to N cycling, and inactivate pesticides and other soil pollutants. Therefore, the loss of Actinobacteria abundance after mineral N application might potentially decrease the soil potential for alleviating abiotic stress in crops [51].

Most of the manures stimulated an increase in the share of Proteobacteria in the soil microbiome. Poultry and rabbit manure resulted in the increase in Bacteroidetes abundance. These phyla are very abundant in human and animal intestines; therefore, manure application might transfer the bacteria to the soil. Larsbrink and McKee [52] noted that Bacteroidetes possess a very efficient energy-saving system enabling good survival in a competitive soil environment. 

Interestingly, a very strong shift in Firmicutes was observed after application of sewage sludge I, which made this phylum the most abundant in the entire bacterial population, whereas in other fertilization variants soils were dominated by Proteobacteria or Acidobacteria. Firmicutes are known to be widely distributed in anaerobic sludge treatment systems and are capable of utilizing a wide range of substrates [53]. Veach et al. [54] reported an increase in abundance of Firmicutes under drought stress in soils, so their wider distribution might result in greater resistance of bacterial communities to reduced moisture.

**Figure 5:**
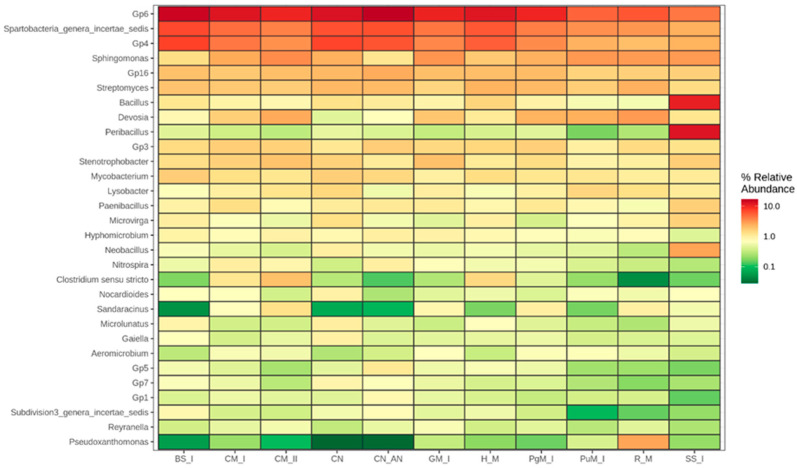
Most abundant genera in soil after application of EOMs (columns from the left: BS—rural bottom sediment; CM—cattle manure; CN—control; CN_AN—control-AN; GM—goat manure; HM—horse manure; PgM—pig manure; PuM—Poultry manure; RM—rabbit manure; SS—sewage sludge I; I—rate 20 t ha^−1^, II—rate 40 t ha^−1^). Not fully recognized Gp group of genera belongs to phylum Acidobacteria.

The relative abundance of the most widely distributed genera are presented in Figure 5. The control soil and soil fertilized with synthetic N exhibited rather similar patterns of genera abundance. It can be only emphasized that ammonia nitrate addition slightly increased the abundance of *Nitrospira,* which is responsible for the nitrification process. This can be attributed to the greater availability of ammonia in soil. When analyzing the rate effect of cattle manure, an increase in *Sphingomonas*, *Devosia*, *Clostridium*, *Sandaracinus* and *Stenotrophobacter* abundance was observed when the rate of 40 t manure ha^−1^ was applied, compared to the lower dose, whereas *Nitrospira* was slightly reduced. *Sphingomonas* has the capacity to fix atmospheric nitrogen [55]. *Devosia* has been known for its capacity for nitrification and denitrification, playing an important role in nitrogen transformation [56]. *Clostridium*, which is commonly found in manure, and has a capacity to fix nitrogen, takes part in fermentation and decomposes cellulose. Therefore, the increased contribution of these genera to the total abundance of bacteria in soil can be rather understood as a positive shift; however, more research is needed to fully understand the importance of such changes. 

Goat manure was stimulative to *Stenotrophobacter*, belonging to Acidobacteria. Rabbit manure greatly enhanced *Pseudoxanthomonas* and to a certain extent *Devosia* abundance. Bottom sediment did not stimulate dramatic changes in genera pattern but slightly stimulated soil richness in *Mycobacteria*. The sewage sludge made a significant shift in *Bacillus*, *Peribacillus* and *Neobacillus*, which become the most abundant genera in the soil.

**Figure 6:**
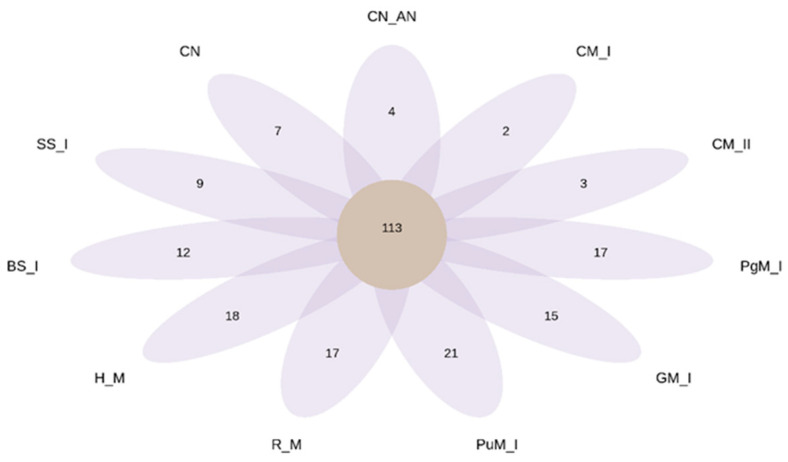
Venn diagram showing the numbers of shared and unique bacterial genera in soil after the tested treatments (Abbreviations: BS—rural bottom sediment; CM—cattle manure; CN—control; CN_AN—control AN; GM—goat manure; HM—horse manure; PgM—pig manure; PuM—Poultry manure; RM—rabbit manure; SS—sewage sludge I; I—rate 20 t ha^−1^, II—rate 40 t ha^−1^). The central circle shows the number of common bacterial genera whereas the other numbers represent unique genera for the given soil treatment

The Venn diagram (Figure 6) provides information on unique and common bacterial genera after soil fertilization. The core soil microbiome consisted of 113 genera. Control soils contained 11 other autochthonic genera that were not detected in EOM-treated soils, likely being displaced by newly introduced bacteria. The largest number of unique genera was found after the application of poultry and horse manure, followed by pig and rabbit manure. Sewage sludge introduced nine new genera to soil that adopted to soil conditions. Interestingly, cattle manure introduced a very small number of new genera (2–3, depending on the manure rate). 

### 2.3. Effect of EOMs on Soil Chemical Properties and Plant Biomass

Even at the lower EOM rate, a significant increase in ammonia-N was observed after the application of goat and poultry manure; however, its levels were rather low in all samples (Table 5). It might have indicated that ammonia were easily converted to nitrates. A greater EOM dose did not result in raising values of ammonia-N contents (Table 6). There were no significant differences between the treatments, except for sewage sludge II, which increased ammonia-N up to 11.7 mg kg^−1^ as compared to <1 for all other variants. The synthetic N fertilizer raised nitrate-N in soil from 18.9 to 60.4 mg kg^−1^, but the greater increase was observed after application of both doses of poultry manure, rabbit manure and both sewage sludges. As shown in Table 5 and Table 6, nitrate-N was high in soil amended with sewage sludge II; therefore, it can be concluded that the observed high ammonia content in this soil was not driven by nitrification inhibition. 

The chemical data for planted soils are not presented here. However, growing plants reduced nitrate content in most cases since they were absorbed by plant roots. In general, the pattern of nitrate contents was similar as in non-planted pots: rabbit manure and both sewage sludges generated greater nitrate availability than the mineral fertilizer. The data indicate a risk of nitrate leaching after application of high rates of poultry, rabbit and horse manure, and even greater risk when sewage sludge is applied, if crops are not planted shortly after the EOM application. 

The soil was initially characterized by medium content of available phosphorus. The average available P in arable soils in Poland is 167 mg P_2_O_5_ kg^−1^ [16]. When the lower EOM rate was applied, both sewage sludges and rabbit manure enabled greater P availability than in control soil (Table 5). Almost all EOMs resulted in the increased P solubility when the greater dose was introduced to the soil (Table 6). As shown in Table 2, the most rich in P were sewage sludge samples, pig manure, horse manure and rabbit manure, and these amendments stimulated the greatest P availability in soil. This proves them to be a good source of P and alternative to phosphate mineral fertilizers. Potassium availability in soil was stimulated by all manure types at greater doses and pig, rabbit, goat and horse manure at the rate of 20 t ha^−1^. This observation, in general, corresponded to K concentration in EOMs. Soil pH values remained stable after EOM application, regardless of the rate used (Table 5 and Table 6). The only exceptions were poultry manure and sewage sludge II that, similarly as the synthetic fertilizer, acidified the soil. This process was especially intensive in the case of the sludge. The greater EOM dose further accelerated this process. N fertilizer-induced soil acidification has been observed also in plot conditions [21], similarly as sludge-induced pH decreases [57]. In our study, this was likely responsible for the increase in Acidobacteria abundance in soil treated with N mineral fertilizer. These soil treatments shall be accompanied by liming practices to counteract the soil degradation through acidification. It is worth noting that the observed pH drop to the slightly acidic range after sewage sludge II application might have stimulated the increase in P availability.

The EOMs additions differentiated the mustard biomass yield (Table 7 and Table 8). Four treatments resulted in the substantial increase in shoot biomass, corresponding to root development; these were poultry manure, rabbit manure and both sewage sludges. The positive short term impact of sewage sludge application on crop yields have been previously reported [58,59]. Referring soil chemical properties to the mustard yields, it can be noted that great plant yields after the poultry manure treatment have explained the observed reduction of nitrates in planted soil. In general, the yield stimulation corresponded to greater nitrate availability in soil (Table 5 and Table 6). We can assume that this was a key factor determining the short-term crop response to EOM application. Only in the case of rural bottom sediment, the greater EOM rate did not increase the shoot yield. Doubling the dose of other EOMs stimulated the increase in shoot yield by 9 to 152% as compared to the 20 t rate. The greatest dose response was observed for rabbit manure (152% increase) and sewage sludge II (38% increase). 

**Figure 7:**
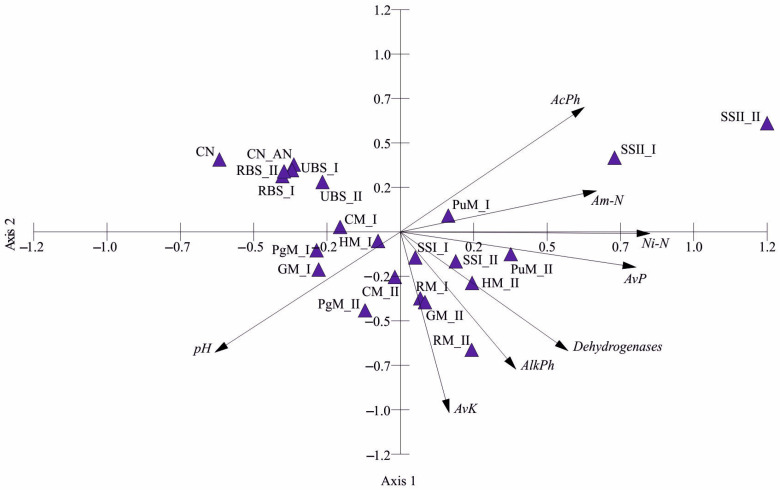
Principal component analysis (PCA) of enzymatic and nutrient parameters across EOM types and rates (EOM abbreviations: RBS—rural bottom sediment; UBS—urban bottom sediment; CM—cattle manure; CN—control; CN AN—control AN; GM—goat manure; HM—horse manure; PgM—pig manure; PuM—Poultry manure; RM—rabbit manure; SSI—sewage sludge I; SSII—sewage sludge II; I—rate 20 t ha^−1^, II—rate 40 t ha^−1^. Parameter abbreviations: AlkPh—alkaline phosphatase; AcPh—acidic phosphatase; AvK—available potassium; AvP—available phosphorus; Ni-N—nitrate nitrogen; Am-N—ammonia nitrogen).

PCA analysis enabled an integrated exploration of EOM effects across EOM types and rates (Figure 7). A similarity in parameter responses to particular EOMs across both rates was commonly observed. This would mean that the impact of EOMs on soil enzymatic activity and nutrient availability is EOM-specific, whereas their rates might accelerate the intensity of the observed responses. The two axes of PCA explained 72.97% of the total variance, which can be sufficient to describe the effects of observed EOMs (Table S6). Axis 1 shows a positive correlation with both phosphatases, dehydrogenases, mineral N, availability of phosphorus and the negative correlation with soil pH. The EOMs supporting high values of nutrient availability and activity of enzymes are clearly separated on the right side of the ordination space. Samples with the lowest values of these parameters are located on the left side. The ANOVA analysis revealed the significant effect of rate on soil enzymes (Figure 1); however, Figure 7 reveals that the pattern of EOM effects is similar at both doses, since both rates of particular EOMs are closely located in the ordination space. 

## 3. Materials and Methods

### 3.1. Experimental Scheme

The greenhouse experiment was carried out in the research station of the Institute of Soil Science and Plant Cultivation—State Research Institute (IUNG-PIB). The experiment consisted of 132 white plastic pots (3 L volume) filled with a loamy sand soil. The organic carbon content in soil was low (0.69%), and initial soil pH was 6.4. 

The soil was treated with a range of EOM types: 10 EOMs were tested, including 6 types of manure (cattle, pig, goat, poultry, rabbit and horse manure); 2 bottom sediments (from urban and rural systems); and 2 types of municipal sewage sludge representing different city sizes. Two control variants were used as a reference: with no fertilization (Control) and fertilized with ammonium nitrate added as solid fertilizer (Control—AN). 

The tested manure batches came from various production farms located in the Lubelskie Voivodeship in Eastern Poland. Bottom sediments used in the pot experiment were collected from ponds located in the urban and rural area in the Lubelskie Voivodeship, and sewage sludge was collected from two municipal wastewater treatment plants, located in the Wielkopolskie Voivodeship and Lubelskie Voivodeship in cities with the number of inhabitants being approx. 8 thousand (sewage sludge I) and 47 thousand (sewage sludge II), respectively. Sewage sludge was collected after it was dehydrated in the press. 

The rates of organic amendments applied to pots are listed in Table 9. Each EOM material was applied at 2 rates, corresponding to 20 and 40 tons of the material per hectare. There were two control variants: fully untreated control and control-AN, which was fertilized with ammonium nitrate in an amount corresponding to 170 kg N per ha. The experimental scheme included 2 different variants of the same fertilization combinations: with and without plants. The latter variant was tested to assess the exclusive effect of fertilization on the biochemical and enzymatic activity and biodiversity of the soil after the application of EOM. Each treatment combination was represented by 3 replications (pots). The applied experimental scheme resulted in 132 pots being used in the experiment. 

1300 g of soil was poured into the pots with the appropriate amount of organic materials, and then the pot contents were thoroughly mixed. Then, the soil in the pots was watered to a moisture corresponding to approx. 60% of the field water capacity. The soil was left for 2 weeks to react with the organic amendments. After this time, plants were sown. The test plant was white mustard (*Sinapis alba* L.), belonging to the Brassicaceae family, due to its rapid emergence and a high biomass production rate. 

After 2 months, when the plants had sufficient biomass, the plants were harvested and weighed fresh and after drying. Subsequently, soil samples were taken from the pots. They were thoroughly mixed so that the material was homogeneous and sieved through a 2 mm sieve to remove plant debris and mixed again. The soil samples for enzymatic and biochemical analyses were stored in closed plastic bags at a temperature of 4 ± 1 °C. Soil samples for physico-chemical analyses were dried and stored in paper bags at a temperature of 20 ± 2 °C. The soil samples intended for diversity analysis were stored at −80 °C. No samples were stored longer than 1 month before being analyzed, since within this time all the chemical and biological analyses were performed. 

The physico-chemical and enzymatic analyses were performed for samples collected from all pots (132 samples). Biochemical analyses using the Biolog EcoPlate system were performed for unplanted soils for all samples corresponding to 20 tons of EOM dry weight and for samples representing the higher dose of the cattle manure to reflect the effect of a dose. In total, 39 samples were subjected to the Biolog EcoPlate analysis. The aim of this analysis was to evaluate the EOM effects on diversity of C sources utilized by soil microorganisms which is indicative of the functional diversity of the microbiome. Next-generation sequencing (NGS) analysis was performed for the 20-tons rate for all manure-treated samples and the selected bottom sediment (rural bottom sediment) and sewage sludge (sewage sludge I). Additionally, samples representing 40 t rate of cattle manure were included in the NGS analysis to reflect the dose effect. This analysis aimed at exploring EOM effects on diversity of bacteria and shifts in abundance of particular phyla or genera. 

In order to ensure the quality of the obtained biological results, the enzymatic activity (dehydrogenases, acid and alkaline phosphatases) and functional activity (assessment of the metabolic profile—Biolog Ecoplate) were performed in 3 technical repetitions for each soil sample collected from pots and subjected to these analyses.

### 3.2. Chemical Analysis of EOM

Total carbon (TC) and total nitrogen (TN) contents in EOM were determined using a Vario Macro Cube CN elemental analyzer (Elementar Analysensysteme GmbH, Langenselbold, Germany) after dry combustion. Total trace element, P and K contents were measured after digestion of a sample in a 3:1 mixture of concentrated HNO3:HCl in Teflon PFA vessels in a microwave-accelerated reaction system (MarsXpress; CEM Corp., Matthews, NC, USA) followed by measurements of the elements in the extracts by ICP-MS (Agilent 7500ce, Agilent Tech., Santa Clara, CA, USA).

The content of potentially toxic organic compounds involved determination of pesticides (PEST) and polycyclic aromatic hydrocarbons (PAHs). PEST analysis included BHC-alpha; BHC-beta, BHC-gamma, BHC-delta; Chlorpyrifos-methyl; Aldrin, Heptachlor, Endosulfan I; Dieldrin; DDE-p,p’, Endrin; Endosulfan II; M-DDD-p,p’, Endosulfan; DDT-p,p’, and Methoxychlor-p,p’, while PAHs analysis covered 16 compounds from the US EPA List, namely Naphthalene-Nap, Acenaphthylene-Acy, Acenaphthene-Ace, Fluorene-Fl, Phenanthrene-Phe, Anthracene-Ant, Fluoranthene-Fla, Pyrene-Pyr, Benzo(a)anthracene-BaA, Chrysene-Chr, Benzo(b)fluoranthene-Bbf, Benzo(k)fluoranthene-BkF, Benzo(a)pyrene-BaP, Indeno(1,2,3-cd)pyrene-IcdP, Dibenz(a, h)anthracene-DahA, and Benzo(ghi)perylene-BghiP. 

All PEST and PAHs compounds were extracted and determined in EOM samples dried at 40 °C following the analytical procedure described by Ukalska-Jaruga et al. [60,61,62]. The PEST and PAHs compounds detection was carried out using gas chromatography triple mass spectrometry on an Agilent 7890B GC system (Agilent Tech., Santa Clara, CA, USA), equipped with an Agilent 7000C detector and Agilent 7693 Autosampler. Sample analysis was performed in multiple reactor monitoring (MRM) mode with diagnostic ions as recommended in ISO 22892:2006. Quality control measures included blank check with each analytical series, analysis of a certified reference material, duplicate matrix samples, and a solvent blank sample. The precision expressed as a relative standard deviation (RSD) was in the range of 3–7%, and the recovery for individual compounds was within 77–92% for PEST and 78–94% for PAHs. The limit of quantification (LoQ) for individual compounds ranged from 0.001–0.05 and 0.02–2.10 µg kg^−1^ while the limit of detection (LoD) fitted within the 0.007–0.08 and 0.01–0.81 µg kg^−1^ range, respectively, for PEST and PAHs.

### 3.3. Microbiological Soil Analysis

#### 3.3.1. Enzymatic Activity

The activity analyses of three enzymes were performed in order to represent overall microbial activity (dehydrogenases) and important processes of phosphorus cycling in soil (acid and alkaline phosphatases). Determination of the alkaline phosphatase and acid phosphatase activity was carried out by the colorimetric method using PNP (sodium p-nitrophenyl phosphate), after 1-h incubation at 37 °C, at a wavelength of 410 nm [63]. Determination of dehydrogenase activity was performed by the colorimetric method with the use of 3% TTC (triphenyltetrazole chloride) as a substrate, after 24-h incubation at 37 °C, at 485 nm [64]. Colorimetric measurements were performed using the Nicolet spectrophotometer (Thermo Fisher Scientific, Waltham, MS, USA).

#### 3.3.2. Microbial Functional Diversity

Evaluation of the functional diversity of soil microbial communities was performed using the Biolog EcoPlate system (Biolog, Hayward, CA, USA). It enabled the determining of the soil metabolic profile (CLPP—community level physiological profile). Biolog ECO plates are 96-well microplates containing 31 different carbon substrates in triplicate and water as a control. Tetrazolium violet is used as the indicator of color change corresponding to utilization of a given carbon substrate by microorganisms. The induction of average well color development (AWCD) indicates the overall metabolic activity of microorganisms in the sample tested. A suspension of soil samples from the pot experiment for inoculation of the wells was prepared according to the following procedure: 1 g of soil was weighed, transferred to sterile microbiological bottles with a capacity of 200 mL containing 99 cm^3^ of sterile 0.9% NaCl. Each bottle containing the suspension was thoroughly mixed on a shaker (150 rpm for 30 min at 25 °C) and then cooled at 4 °C for 30 min according to the appropriate protocol [65]. A multichannel pipette with a capacity of 120 µL was used and this volume was applied to each well. The plates were incubated in the dark at 28 °C. The results were recorded every 24 h for 7 days, and the results presented in the publication correspond to the time 144 h at absorbance 590. This seems to be the optimal time to analyze the data, as AWCD tended to stabilize [66,67].

#### 3.3.3. NGS—Next Generation Sequencing

Composite samples, representing EOM-treated soils and controls, were prepared for analysis of bacterial diversity. Parts of homogenous replicates (approx. 50 g) were pooled together, homogenized and, subsequently, 0.35 g samples were subjected to the DNA extraction. Total DNA was extracted from soil samples using the FastDNA™ SPIN Kit for soil (MPBiomedicals LLC, Irvine, CA, USA) according to the manufacturer’s specification, and the V3–V4 region of the 16S rRNA gene was sequenced using 341F and 785R primers [68] in 2 bp × 300 bp paired-end technology using the Illumina MiSeq system (Illumina Inc., San Diego, CA, USA). Demultiplexed fastq files were processed using the DADA2 (1.12) package [69] in R software (3.4.3) [70]. Forward and reverse reads were trimmed according to the results obtained from the quality analysis, and primer sequences were removed from all reads. The filtering parameters were as follows: maxN = 0, maxEE for both reads = 2, truncQ = 2. MaxEE corresponds to the maximum expected errors. Error rates were estimated by learnErrors using one billion reads. Sequences were de-replicated using derepFastq with default parameters, and the exact sequence variants were resolved using dada. RemoveBimeraDenovo then was used to remove chimeric sequences. Taxonomy was assigned against the latest version of the RDP database using IDTAXA [71]. The results were converted and imported into the phyloseq (1.22.3) package [72]. Sequences belonging to the chloroplast or mitochondrial DNA were removed. Subsequently, for further analysis, the total number of reads for the individual taxa was converted to a percentage, assuming the sum of all taxa in the individual samples as 100%. Alpha and beta diversity indexes, Venn analysis, RDA analysis, and graphs for taxonomy abundance were prepared in R v.3.4.3 using the microeco package (v.0.7.1) [73].

### 3.4. Chemical Soil Analysis

The contents of ammonium, nitrite and nitrate nitrogen were determined by continuous flow analysis with segmented flux and spectrophotometric detection on a flow analyzer QuAAtro39 (Seal Analytical GmbH, Norderstedt, Germany) after extraction with 1 M K_2_SO_4_ solution in a weight/volume ratio 1:10 (ISO 13395: 1996. Water quality—Determination of nitrite nitrogen and nitrate nitrogen and the sum of both by flow analysis (CFA and FIA) and spectrometric detection; ISO 11732: 2005. Water quality—Determination of ammonium nitrogen—Method by flow analysis (CFA and FIA) and spectrometric detection). The available phosphorus (P) was measured by an Egner–Riehm colorimetric method with extraction of calcium lactate (0.02 M) in dilute HCl (0.01 M) followed by a colorimetric measurement on a Perkin Elmer Lambda 45 Spectrometer (Perkin Elmer, Waltham, MA, USA) based on the reaction with ammonium molybdate. The available potassium (K) was measured after the same AAS extraction using AAnalyst 800 (Perkin Elmer, Waltham, MA, USA). Soil pH was measured in a water suspension with a combined glass electrode with a soil/water ratio 1:2.

### 3.5. Statistical Analysis

The results were statistically analyzed using Statistica v.13.0 software (TIBCO Software Inc. Palo Alto, CA, USA). Three-way ANOVA was performed for testing the main effects and the interactions between the three independent variables: EOM type, rate and the presence of plants. This analysis was performed for the activity of soil enzymes: acidic phosphatase, alkaline phosphatase and dehydrogenases, which were measured for all pot samples. In order to compare the impact of EOMs, the results were expressed as means, and the differences were considered significant at *p* < 0.05. The difference between the variants was identified by ANOVA and Tukey’s test. Indicators of microbial functional diversity from Biolog Ecoplate, such as AWCD and Shannon index (H’), substrate evenness (E’) and substrate richness (R), were calculated for data after 144 h. Principal component analysis (PCA) was applied to explain the relationships between the studied enzymatic and chemical parameters and to show the variability of factors, taking the EOM rate into account. Prior to the PCA, the data were centered and log-transformed. The analyses were conducted using the statistical package (MVSP) program version 3.1 [74]. For analysis of statistical differences between NGS-derived abundance of phyla or genera, we used the Metastats approach [75]. The analysis revealed statistically significant differences for most of the abundance comparisons at phyla and genera levels.

## 4. Conclusions

The study confirmed the hypothesis that various manure types and other EOMs might significantly differentiate responses of the soil microbiome. These responses might have been stimulated by EOM effects on soil pH and nutrient availability and changes in the structure of microbial populations. Substantial shifts in microbiome structure were observed especially after application of poultry and rabbit manure, and one of the tested sewage sludges. EOMs deliver genera to soil that are characteristic of their microbiome, and some of these genera are apparently able to persist in soil. These shifts might have also resulted in alteration of the microbial functional diversity and capability since the degree of utilization of given carbon substrates varied depending on the applied EOM.

Manure did not exhibit any negative effects on microbial parameters, regardless of manure type, even if shifts in functional variability of C utilization were observed. The overall functional diversity was improved by cattle and goat manure. It is worth noting that even the rate of manure corresponding to 20 tons per hectare stimulates soil enzymes. 

Bottom sediments did not exhibit effectiveness in improving plant growth or soil biodiversity and biological activity. These might have been related to lower contents of organic carbon and nutrients in the sediments or the presence of various types of contaminants. 

In general, sewage sludge resulted in no harm to soil microbial parameters even at a rate of 20 t ha^−1^. This means that the permissible rate of municipal sludge to be utilized in agriculture in Poland (3 t annually) can be potentially increased without harm to soil microbial activity and biodiversity if the sludge meets the quality criteria.

EOMs differentiate short-term plant growth, which in our study to large extent was attributed to the diversified rate of nitrate release. Manure and sewage sludge appeared to be an efficient source of nutrients even from the short-term perspective. Nutrient availability for crops very well reflects the total contents of nutrients in EOMs. 

The effect of raising the EOM rate from 20 to 40 t ha^−1^ on biological parameters was EOM-specific. A higher dose of some types of manure resulted in a further increase in activity, while other types of EOM showed no significant effect or even a decrease in activity. It can be therefore stated that the dose equivalent to 20 tons per hectare is sufficient from the perspective of soil biological processes. It must be also emphasized that our study does not refer to risk of pathogen contamination, which is another EOM quality criterion to be taken into account when applying various EOMs to soil. 

## Figures and Tables

**Table 1:** Chemical characteristics of EOM materials tested in the pot experiment.

EOM	Carbon	The Content of Nutrients				Trace Metal Content		
		Nitrogen	Phosphorus	Potassium	Zinc	Lead	Cadmium	Chromium
	[%]				[mg kg^−1^]			
Cattle manure	41.8	2.06	0.62	5.37	147	1.6	0.35	4.7
Pig manure	30.1	2.26	1.62	7.18	195	5.6	0.37	21.4
Goat manure	38.5	2.35	0.99	5.75	93	2.1	0.25	9.9
Poultry manure	17.6	1.75	0.74	1.04	342	86.6	0.63	63.1
Rabbit manure	37.1	3.25	1.36	7.62	206	2.4	0.45	10.3
Horse manure	35.9	2.43	1.29	4.18	121	2.3	0.32	10.2
Rural bottom sediment	1.2	0.07	0.03	0.09	7	4.8	0.07	5.7
Urban bottom sediment	1.9	0.10	0.03	0.03	65	17.7	0.22	21.2
Sewage sludge I	27.3	3.55	2.74	0.36	344	11.1	0.53	96.4
Sewage sludge II	32.7	5.24	1.28	0.23	1309	16.8	1.06	65.4

**Table 2:** Summary of three-way analysis of variance (ANOVA) testing the effects of EOM type, rate and presence of plants.

Variables		Acid Phosphatase Activity		Alkaline Phosphatase Activity		Dehydrogenases Activity	
		*F*	*p*	*F*	*p*	*F*	*p*
Main effects	EOM	60.097	<0.001	20.679	<0.001	23.451	<0.001
	rate	15.836	<0.001	28.777	<0.001	31.653	<0.001
	plant	82.240	<0.001	97.576	<0.001	168.125	<0.001
Interaction effects	EOM × rate	6.317	<0.001	2.051	0.044	3.903	<0.001
	EOM × plant	12.153	<0.001	1.614	0.125	8.248	<0.001
	rate × plant	16.352	<0.001	4.683	0.033	8.329	0.005
	EOM × rate × plant	5.746	<0.001	1.071	0.393	0.995	0.450

**Table 3:** Enzymatic activity in the non-planted soil as an effect of exogenous organic matter addition as dependent on EOM type and their rates.

Soil Additive	Rate [t ha^−1^]	Acid Phosphatase Activity	Alkaline Phosphatase Activity	Dehydrogenase Activity
		[µg PNP g d.m.^−1^ h^−1^]	[µg PNP g d.m.^−1^ h^−1^]	[TTC g d.m.^−1^ h^−1^]
Control		43.8 ^b B^ * ± 2.1	53.5 ^ac B^ ± 5.5	9.5 ^b C^ ± 1.1
Control (ammonium nitrate)		44.7 ^b B^ ± 3.2	56.0 ^ac B^ ± 8.9	9.8 ^b C^ ± 2.3
Cattle manure	20	49.9 ^b^ ± 4.6	80.6 ^abc^ ± 25.8	28.3 ^bc^ ± 9.3
	40	47.9 ^B^ ± 3.0	91.3 ^AB^ ± 28.8	36.7 ^ABC^ ± 6.7
Pig manure	20	43.9 ^b^ ± 0.9	60.4 ^abc^ ± 5.3	23.9 ^bc^ ± 10.3
	40	35.2 ^B^ ± 4.9	73.6 ^AB^ ± 5.5	30.9 ^BC^ ± 5.4
Goat manure	20	40.6 ^b^ ± 6.5	78.3 ^abc^ ± 11.2	30.6 ^abc^ ± 11.4
	40	48.9 ^B^ ± 6.6	91.8 ^AB^ ± 17.8	53.3 ^ABC^ ± 13.3
Poultry manure	20	50.6 ^b^ ± 8.3	94.8 ^b^ ± 9.7	22.4 ^bc^ ± 1.7
	40	46.5 ^B^ ± 4.3	127.1 ^A^ ± 13.4	24.3 ^BC^ ± 2.8
Rabbit manure	20	41.1 ^b^ ± 0.8	88.0 ^ab^ ± 11.4	30.9 ^abc^ ± 5.4
	40	42.2 ^B^ ± 10.8	87.3 ^AB^ ± 36.4	44.7 ^ABC^ ± 17.4
Horse manure	20	50.7 ^b^ ± 6.8	62.9 ^abc^ ± 12.8	37.3 ^ac^ ± 14.4
	40	52.8 ^B^ ± 8.5	73.4 ^AB^ ± 15.1	76.5 ^A^ ± 17.3
Rural bottom sediment	20	48.1 ^b^ ± 8.5	45.5 ^c^ ± 2.4	18.8 ^bc^ ± 1.4
	40	50.3 ^B^ ± 3.4	47.6 ^B^ ± 11.7	15.4 ^BC^ ± 4.2
Urban bottom sediment	20	49.3 ^b^ ± 0.2	43.9 ^c^ ± 10.4	17.5 ^bc^ ± 2.1
	40	51.2 ^B^ ± 2.3	54.2 ^B^ ± 6.3	16.3 ^BC^ ± 3.6
Sewage sludge I	20	48.6 ^b^ ± 1.2	96.2 ^b^ ± 22.5	36.4 ^ac^ ± 2.6
	40	44.2 ^B^ ± 6.6	86.7 ^AB^ ± 33.2	28.9 ^BC^ ± 2.0
Sewage sludge II	20	74.8 ^a^ ± 11.9	58.8 ^abc^ ± 5.9	50.9 ^a^ ± 3.4
	40	77.5 ^B^ ± 11.5	66.7 ^B^ ± 11.8	34.0 ^BC^ ± 14.9

* Means marked with the same lowercase letter did not differ significantly across the treatments (*p* < 0.05, *n* = 3) at the rate of 20 t ha^−1^, according to the Tukey test. Means marked with the same capital letter did not differ significantly across the treatments at the rate of 40 t ha^−1^.

**Table 4:** Changes in microorganism metabolic diversity in soils as expressed by Shannon diversity index (H’), Evenness (E) and substrate richness (R), as calculated after 144 h incubation of EcoPlates.

Treatment	H’	E	R
Control	3.295 ^ab^ * ± 0.035	0.982 ^ab^ ± 0.005	28.667 ^ab^ ± 0.577
Control (ammonium nitrate)	3.338 ^abc^ ± 0.027	0.985 ^ab^ ± 0.007	29.667 ^ab^ ± 1.155
Cattle manure, I Rate	3.392 ^c^ ± 0.015	0.991 ^ab^ ± 0.006	30.667 ^b^ ± 0.577
Cattle manure, II Rate	3.385 ^c^ ± 0.011	0.989 ^ab^ ± 0.002	30.667 ^b^ ± 0.577
Pig manure, I rate	3.374 ^bc^ ± 0.008	0.986 ^ab^ ± 0.006	30.667 ^b^ ± 0.557
Goat manure, I rate	3.387 ^c^ ± 0.018	0.989 ^ab^ ± 0.007	30.667 ^b^ ± 0.557
Poultry manure, I rate	3.378 ^bc^ ± 0.015	0.993 ^ab^ ± 0.004	30.000 ^ab^ ± 0.000
Rabbit manure, I rate	3.379 ^bc^ ± 0.013	0.987 ^ab^ ± 0.002	30.667 ^b^ ± 0.057
Horse manure, I rate	3.312 ^abc^ ± 0.021	0.984 ^ab^ ± 0.005	29.000 ^ab^ ± 1.000
Urban bottom sediment, I rate	3.258 ^a^ ± 0.051	0.978 ^a^ ± 0.005	28.000 ^ab^ ± 1.732
Rural bottom sediment, I rate	3.322 ^abc^ ± 0.036	0.980 ^ab^ ± 0.003	29.667 ^ab^ ± 1.155
Sewage sludge I, I rate	3.339 ^abc^ ± 0.026	0.979 ^a^ ± 0.003	30.333 ^ab^ ± 0.577
Sewage sludge II, I rate	3.292 ^ab^ ± 0.056	0.992 ^ab^ ± 0.007	27.667 ^a^ ± 1.528

* Means marked with the same letter did not differ significantly across the treatments (*p* < 0.05, *n* = 3) according to the Tukey test.

**Table 5:** The effect of EOM on soil chemical parameters, non-planted soil, rate 20 t ha^−1.^

Treatment	Ammonia-N	Nitrite-N	Nitrate-N	Available P (P_2_O_5_)	Available K (K_2_O)	Soil pH
	mg kg^−1^					
Control	0.33 ^abc^ * ± 0,05	0.06 ^a^ ± 0.02	18.9 ^a^ ± 5.33	196 ^a^ ± 70.9	113 ^b^ ± 14.49	7.23 ^b^ ± 0.06
Control—AN	0.08 ^a^ ± 0.02	0.04 ^a^ ± 0.01	60.4 ^ab^ ± 19.71	212 ^a^ ± 4.28	168 ^a^ ± 9.87	6.83 ^a^ ± 0.15
Cattle manure	0.30 ^abc^ ± 0.06	0.05 ^a^ ± 0.01	37.7 ^ab^ ± 2.03	199 ^a^ ± 4.62	237 ^a^ ± 3.21	7.17 ^b^ ± 0.06
Pig manure	0.42 ^bc^ ± 0.31	0.09 ^a^ ± 0.02	29.4 ^a^ ± 1.96	268 ^abc^ ± 16.38	516 ^d^ ± 39.36	7.37 ^b^ ± 0.06
Goat manure	0.84 ^d^ ± 0.13	0.07 ^a^ ± 0.01	28.2 ^a^ ± 0.91	198 ^a^ ± 41.61	478 ^cd^ ± 24.29	7.30 ^b^ ± 0.00
Poultry manure	0.59 ^cd^ ± 0.04	0.12 ^a^ ± 0.03	111.5 ^c^ ± 4.37	281 ^abc^ ± 6.43	234 ^a^ ± 5.19	6.70 ^a^ ± 0.00
Rabbit manure	0.34 ^abc^ ± 0.14	0.15 ^a^ ± 0.02	94.4 ^c^ ± 14.37	344 ^c^ ± 47.16	811 ^e^ ± 51.62	7.13 ^b^ ± 0.12
Horse manure	0.12 ^ab^ ± 0.03	0.14 ^a^ ± 0.10	46.8 ^ab^ ± 16.81	291 ^abc^ ± 34.85	390 ^c^ ± 100.10	7.17 ^b^ ± 0.06
Urban bottom sediment	0.10 ^ab^ ± 0.07	0.19 ^a^ ± 0.16	32.7 ^ab^ ± 6.96	226 ^ab^ ± 4.15	173 ^ab^ ± 36.7	7.17 ^b^ ± 0.06
Rural bottom sediment	0.08 ^a^ ± 0.05	0.06 ^a^ ± 0.02	38.6 ^ab^ ± 6.64	234 ^ab^ ± 0.07	179 ^ab^ ± 2.06	7.13 ^b^ ± 0.06
Sewage sludge I	0.35 ^abc^ ± 0.08	0.06 ^a^ ± 0.00	112.8 ^c^ ± 4.19	301 ^bc^ ± 61.64	182 ^ab^ ± 5.12	7.60 ^b^ ± 0.00
Sewage sludge II	0.35 ^abc^ ± 0.08	0.09 ^a^ ± 0.03	233.1 ^d^ ± 12.45	518 ^d^ ± 60.90	199 ^ab^ ± 28.2	6.10 ^a^ ± 0.10

* Means marked with the same letter did not differ significantly across the treatments (*p* < 0.05, *n* = 3) according to the Tukey test.

**Table 6:** The effect of EOM on soil chemical parameters, non-planted soil, rate 40 t ha^−1.^

Treatment	Ammonia-N	Nitrite-N	Nitrate-N	Available P (P_2_O_5_)	Available K (K_2_O)	Soil pH
	mg kg^−1^					
Control	0.33 ^abc^ * ± 0.05	0.06 ^a^ ± 0.02	18.9 ^a^ ± 5.33	196 ^a^ ± 70.9	113 ^b^ ± 14.49	7.23 ^b^ ± 0.06
Control—AN	0.08 ^a^ ± 0.02	0.04 ^a^ ± 0.01	60.4 ^ab^ ± 19.71	212 ^a^ ± 4.28	168 ^a^ ± 9.87	6.83 ^a^ ± 0.15
Cattle manure	0.30 ^a^ ± 0,09	0.08 ^a^ ± 0.01	66.1 ^ab^ ± 11.57	229 ^ab^ ± 19.95	574 ^b^ ± 81.43	7.17 ^b^ ± 0.06
Pig manure	0.92 ^a^ ± 0.16	0.09 ^a^ ± 0.00	38.5 ^a^ ± 6.20	403 ^cd^ ± 18.99	798 ^d^ ± 75.13	7.50 ^b^ ± 0.10
Goat manure	0.64 ^a^ ± 0.11	0.10 ^a^ ± 0.01	47.9 ^ab^ ± 12.04	303 ^abc^ ± 36.90	719 ^cd^ ± 84.87	7.40 ^b^ ± 0.10
Poultry manure	0.65 ^a^ ± 0.34	0.20 ^a^ ± 0.03	159.7 ^cd^ ± 8.67	355 ^bcd^ ± 10.28	395 ^e^ ± 62.16	6.43 ^a^ ± 0.06
Rabbit manure	0.36 ^a^ ± 0.14	0.15 ^a^ ± 0.02	103.9 ^bc^ ± 9.54	581 ^ef^ ± 79.94	1653 ^f^ ± 58.94	7.57 ^b^ ± 0.21
Horse manure	0.10 ^a^ ± 0.00	0.09 ^a^ ± 0.01	73.3 ^ab^ ± 11.66	388 ^d^ ± 19.86	602 ^bc^ ± 29.54	7.07 ^b^ ± 0.06
Urban bottom sediment	0.11 ^a^ ± 0.03	0.07 ^a^ ± 0.02	33.0 ^a^ ± 6.53	236 ^ab^ ± 04.78	172 ^a^ ± 03.97	7.23 ^b^ ± 0.06
Rural bottom sediment	0.11 ^a^ ± 0.02	0.04 ^a^ ± 0.00	34.5 ^a^ ± 1.91	300 ^abc^ ± 96.76	188 ^a^ ± 13.30	7.13 ^b^ ± 0.06
Sewage sludge I	0.74 ^a^ ± 0.18	0.09 ^a^ ± 0.10	170.9 ^d^ ± 21.7	480 ^de^ ± 33.22	209 ^a^ ± 51.16	7.63 ^b^ ± 0.06
Sewage sludge II	11.72 ^b^ ± 8.20	1.58 ^a^ ± 0.71	295.2 ^e^ ± 58.04	706 ^f^ ± 58.54	202 ^a^ ± 10.50	5.87 ^a^ ± 0.21

* Means marked with the same letter did not differ significantly across the treatments (*p* < 0.05, *n* = 3) according to the Tukey test.

**Table 7:** The effect of EOM on white mustard biomass (g pot^−1^)—rate 20 t ha^−1.^

	Roots	Shoots	Total Biomass
Control	0.82 ^a^ * ± 0.3	4.68 ^a^ ± 1.7	5.50 ^a^ ± 3.0
Control—AN	0.89 ^a^ ± 0.4	5.09 ^a^ ± 2.6	5.98 ^a^ ± 3.1
Cattle manure	0.82 ^a^ ± 0.1	4.79 ^a^ ± 1.1	5.61 ^a^ ± 1.2
Pig manure	0.99 ^a^ ± 0.1	6.15 ^a^ ± 1.1	7.14 ^ab^ ± 1.2
Goat manure	0.73 ^a^ ± 0.3	4.64 ^a^ ± 1.7	5.37 ^a^ ± 2.0
Poultry manure	3.55 ^c^ ± 0.6	25.12 ^b^ ± 0.8	28.67 ^c^ ± 1.2
Rabbit manure	1.67 ^ab^ ± 0.4	13.52 ^c^ ± 3.9	15.19 ^b^ ± 4.3
Horse manure	1.20 ^a^ ± 0.2	6.12 ^a^ ± 0.9	7.32 ^a^ ± 1.1
Urban bottom sediment	0.96 ^a^ ± 0.1	4.64 ^a^ ± 1.0	5.59 ^a^ ± 1.1
Rural bottom sediment	1.07 ^a^ ± 0.0	5.56 ^a^ ± 0.9	6.64 ^a^ ± 0.9
Sewage sludge I	2.83 ^bc^ ± 0.7	24.82 ^b^ ± 2.7	25.72 ^c^ ± 4.3
Sewage sludge II	2.94 ^bc^ ± 0.9	36.82 ^d^ ± 4.0	39.77 ^d^ ± 4.4

* Means marked with the same letter did not differ significantly across the treatments (*p* < 0.05, *n* = 3) according to the Tukey test.

**Table 8:** The effect of EOM on white mustard biomass (g pot^−1^)—rate 40 t ha^−1.^

	Roots	Shoots	Total Biomass
Control	0.82 ^a^ * ± 0.3	4.68 ^a^ ± 1.7	5.50 ^a^ ± 3.0
Control—AN	0.89 ^a^ ± 0.4	5.09 ^a^ ± 2.6	5.98 ^a^ ± 3.1
Cattle manure	0.95 ^a^ ± 0.2	6.01 ^a^ ± 1.9	6.97 ^a^ ± 2.1
Pig manure	0.87 ^a^ ± 0.1	6.96 ^a^ ± 2.3	7.83 ^a^ ± 2.4
Goat manure	0.82 ^a^ ± 0.3	5.60 ^a^ ± 1.3	6.42 ^a^ ± 1.6
Poultry manure	2.91 ^b^ ± 0.1	31.01 ^b^ ± 3.0	33.92 ^b^ ± 3.0
Rabbit manure	3.44 ^b^ ± 0.4	34.13 ^b^ ± 7.2	37.57 ^b^ ± 6.9
Horse manure	1.12 ^a^ ± 0.1	6.67 ^a^ ± 1.3	7.79 ^a^ ± 1.4
Urban bottom sediment	0.84 ^a^ ± 0.3	3.85 ^a^ ± 0.9	4.69 ^a^ ± 1.1
Rural bottom sediment	1.16 ^a^ ± 0.1	6.70 ^a^ ± 1.1	7.86 ^a^ ± 1.2
Sewage sludge I	2.87 ^b^ ± 0.6	27.28 ^b^ ± 1.2	30.15 ^b^ ± 1.7
Sewage sludge II	3.95 ^b^ ± 1.3	50.89 ^c^ ± 3.7	54.83 ^c^ ± 4.7

* Means marked with the same letter did not differ significantly across the treatments (*p* < 0.05, *n* = 3) according to the Tukey test.

**Table 9:** The scheme of the greenhouse experiment and the applied rates of EOM dry matter.

EOM	Per Hectare Equivalent (t)	Per Pot (g pot^−1^)
Control without N fertilizer	0	0
Control with ammonium nitrate	0	0
Cattle manure	20	13
	40	26
Pig manure	20	13
	40	26
Goat manure	20	13
	40	26
Poultry manure	20	13
	40	26
Rabbit manure	20	13
	40	26
Horse manure	20	13
	40	26
Bottom sediment rural	20	13
	40	26
Bottom sediment urban	20	13
	40	26
Sewage sludge I	20	13
	40	26
Sewage sludge II	20	13
	40	26

## Data Availability

The data is not shared due to privacy issues—samples of most EOM originate from private companies.

## Supplementary Materials

The supporting information can be downloaded at: https://www.mdpi.com/article/10.3390/ijms241914559/s1.
