# Supplementary material for: Microbial Community Response to Various Types of Exogenous Organic Matter Applied to Soil
Source: Int J Mol Sci. 2023 Sep 26;24(19):14559. doi: 10.3390/ijms241914559 (PMC10572811; doi:10.3390/ijms241914559)
Supplement: Supplementary file 1 [file ijms-24-14559-s001.zip › Supplementary_tables_S1-6.pdf]

Table S1. Pesticide contents in soil additives tested in the pot experiment

EOM	Pesticides (µg kg^{-1})															
	A	B	C	D	E	F	G	H	I	J	K	L	M	N	O	P
Cattle manure	<LOD	<LOD	<LOD	<LOD	455.24	<LOD	<LOD	<LOD	<LOD	<LOD	<LOD	<LOD	0.09	<LOD	<LOD	<LOD
Pig manure	<LOD	<LOD	<LOD	<LOD	238.63	<LOD	<LOD	<LOD	<LOD	<LOD	<LOD	<LOD	2.39	<LOD	<LOD	<LOD
Goat manure	<LOD	<LOD	<LOD	<LOD	201.21	<LOD	<LOD	<LOD	<LOD	<LOD	<LOD	<LOD	1.10	<LOD	<LOD	<LOD
Poultry manure	<LOD	<LOD	<LOD	<LOD	100.66	<LOD	<LOD	<LOD	<LOD	22.87	<LOD	<LOD	9.53	<LOD	30.22	23.90
Rabbit manure	<LOD	<LOD	<LOD	<LOD	110.56	<LOD	24.22	<LOD	<LOD	<LOD	<LOD	<LOD	0.38	<LOD	<LOD	<LOD
Horse manure	<LOD	<LOD	<LOD	<LOD	101.80	<LOD	<LOD	<LOD	<LOD	<LOD	<LOD	<LOD	0.07	<LOD	<LOD	10.61
Bottom sediment rural	<LOD	<LOD	<LOD	<LOD	110.22	<LOD	<LOD	<LOD	<LOD	<LOD	<LOD	<LOD	0.16	<LOD	<LOD	<LOD
Bottom sediment urban	<LOD	<LOD	<LOD	<LOD	164.49	<LOD	<LOD	<LOD	<LOD	<LOD	<LOD	<LOD	0.37	<LOD	<LOD	16.71
Sewage sludge I	<LOD	<LOD	<LOD	<LOD	180.22	<LOD	7.74	<LOD	<LOD	<LOD	<LOD	<LOD	0.05	<LOD	<LOD	81.34
Sewage sludge II	137.37	102.86	111.94	129.99	616.29	148.36	550.12	552.59	322.08	392.65	385.00	306.42	122.20	272.16	148.09	241.19

A - BHC-alpha; B - BHC-beta, C - BHC-gamma, D - BHC-delta; E - Chlorpyrifos-methyl; F – Aldrin, G - Heptachlor , H – Endosulfan I; I – Dieldrin; J - DDE-p,p' , K – Endrin; L - Endosulfan II; M - DDD-p,p' , N – Endosulfan; O - DDT-p,p' , P - Methoxychlor- p,p'

Table S2. Contents of polycyclic aromatic hydrocarbons in soil additives tested in the pot experiment

EOM	Polycyclic Aromatic Hydrocarbons (μg kg^{-1})															
	A	B	C	D	E	F	G	H	I	J	K	L	M	N	O	P
Cattle manure	127.84	116.34	118.68	129.82	164.29	124.10	136.93	157.10	113.55	111.37	112.76	99.21	102.48	155.51	150.54	152.24
Pig manure	102.77	87.69	91.39	95.74	197.86	108.69	257.69	245.46	188.41	196.53	166.81	207.34	93.68	198.24	255.84	178.19
Goat manure	110.02	99.05	99.98	110.12	145.55	105.97	132.30	180.21	104.35	106.01	123.34	104.61	87.95	137.03	123.94	133.69
Poultry manure	241.51	88.27	177.50	146.09	876.96	266.30	1661.7	1412.6	1243.1	1191.1	1386.6	1158.0	316.9	1093.9	295.65	971.86
Rabbit manure	97.03	87.54	89.70	95.08	138.09	98.47	137.85	138.61	105.77	107.86	99.38	95.83	81.13	129.27	114.22	125.34
Horse manure	112.00	95.97	99.15	105.64	129.53	103.33	121.59	127.49	101.79	102.87	99.88	88.60	85.89	133.79	120.55	130.92
Bottom sediment rural	101.30	93.03	93.68	98.70	142.23	114.78	243.97	228.79	182.66	178.80	175.35	153.15	99.78	162.87	123.60	150.16
Bottom sediment urban	137.37	102.86	111.94	129.99	616.29	148.36	550.12	552.59	322.08	392.65	385.00	306.42	122.20	272.16	148.09	241.19
Sewage sludge I	173.76	113.20	132.94	277.14	128.08	206.34	120.17	610.60	232.66	194.91	193.31	158.45	96.47	193.39	237.05	201.86
Sewage sludge II	489.27	94.20	112.67	212.90	350.96	149.46	272.38	353.36	170.13	228.51	211.18	198.04	98.13	178.92	212.72	181.93

A – Naphthalene; B - Acenaphthylene ; C – Acenaphthene; D – Fluorene; E – Phenanthrene; F – Anthracene; G – Fluoranthene; H - Pyrene; I - Benz[a]anthracene; J - Chrysene; K Benzo[b]fluoranthene, L - Benzo[k]fluoranthene; M - Benzo[a]pyrene, N - Indeno[1,2,3-cd]pyrene ; 0 - Dibenz[a,h]anthracene, P - Benzo[g,h,i]perylene

Table S3. Enzymatic soil activity in planted soil as an effect of exogenous organic matter addition – rate 20 t ha$^{-1.}$

Soil additive	Acidic phosphatase activity	Alkaline phosphatase activity	Dehydrogenases activity
	[μg PNP g d.m.$^{-1}$ h^{-1}]	[μg PNP g d.m.$^{-1}$ h^{-1}]	[TTC g d.m.$^{-1}$ h^{-1}]
Control	58.6^{b*}±6.5	86.7±7.2	42.9±3.0
Control - AN	60.1^{b}±8.3	89.3^{bc}±8.9	41.8^{abcd}±12.0
Cattle manure	55.3^{b}±4.5	95.3^{ab}±4.4	62.37^{ab}±7.1
Pig manure	52.1^{b}±0.5	88.8^{bc}±7.3	51.8^{abcd}±10.4
Goat manure	51.3^{b}±6.3	96.6^{ab}±11.1	69.4^{a}±21.1
Poultry manure	59.3^{b}±4.5	118.8^{a}±4.5	73.9^{a}±8.6
Rabbit manure	49.3^{b}±3.9	100.1^{ab}±8.8	57.3^{abc}±19.9
Horse manure	54.8^{b}±3.2	84.1^{bc}±9.2	62.8^{ab}±2.6
Rural bottom sediment	49.0^{b}±2.9	80.1^{bc}±2.4	27.1^{cd}±4.3
Urban bottom sediment	53.7^{b}±0.7	69.7^{c}±5.6	20.6d±7.1
Sewage sludge I	45.9^{b}±3.1	94.9^{ab}±14.8	33.93^{bcd}±2.9
Sewage sludge II	93.5^{a}±12.6	98.9^{ab}±10.1	73.5^{a}±8.3

*Means marked with the same letter did not differ significantly across the treatments ($p < 0.05$, $n = 3$) according to the Tukey test

Table S4. Enzymatic soil activity in planted soil as an effect of exogenous organic matter addition – rate 40 t ha^{-1}

Soil additive	Acidic phosphatase activity	Alkaline phosphatase activity	Dehydrogenases activity
	[µg PNP g $d.m.^{-1}$ h^{-1}]	[µg PNP g $d.m.^{-1}$ h^{-1}]	[TTC g $d.m.^{-1}$ h^{-1}]
Control	61.2^{bc*}±6.5	91.3^{cd}±7.2	43.5^{bcd}±3.0
Control -AN	60.1^{bc}±8.3	89.3^{cd}±8.9	41.8^{bcd}±12.0
Cattle manure	59.9^{bc}±6.0	108.7^{bcd}±6.3	92.2^{a}±27.7
Pig manure	50.9^{c}±5.3	101.3^{cd}±1.8	85.6^{ad}±6.0
Goat manure	48.95^{c}±2.9	115.1^{bcd}±6.9	92.4^{a}±13.7
Poultry manure	81.1^{b}±5.7	173.9^{a}±34.3	108.9^{a}±7.8
Rabbit manure	65.1^{bc}±21.8	153.5^{ab}±32.8	76.2^{acd}±25.2
Horse manure	59.9^{bc}±4.9	104.1^{cd}±7.3	105.7^{a}±23.2
Rural bottom sediment	51.7^{c}±1.5	74.5^{d}±1.0	21.5^{b}±3.2
Urban bottom sediment	50.2^{c}±3.4	74.3^{d}±7.5	22.4^{b}±4.7
Sewage sludge I	46.7^{c}±4.4	109.4^{bcd}±10.0	32.4^{bc}±5.3
Sewage sludge II	156.6^{a}±18.0	124.6^{bc}±8.1	93.9^{a}±15.1

*Means marked with the same letter did not differ significantly across the treatments ($p < 0.05$, $n = 3$) according to the Tukey test

Table S5. Percentage contribution of C source groups in total C utilization by microorganisms after addition of EOMs to soil

Treatment	Amines and amides	Amino acids	Carbohydrates	Carboxylic acids	Polymers
Control	2.39^{a*}±1.1	19.13^{a}±4.5	32.01^{cd}±1.6	30.53^{a}±3.7	15.93^{e}±3.0
Control (ammonium nitrate)	5.95^{ab}±2.7	21.62^{a}±0.9	31.16^{bcd}±1.3	31.22^{a}±3.1	10.05^{abc}±1.0
Cattle manure, I Rate	5.86 ^{ab}±1.9	19.24^{a}±1.4	28.42^{abc}±1.2	33.09^{a}±1.2	13.39^{bcde}±1.1
Cattle manure, II Rate	7.89^{b}±0.4	20.24^{a}±2.1	27.77^{ab}±1.2	31.42^{a}±1.7	12.69^{bcde}±0.9
Pig manure, I rate	6.11 ^{ab}±0.7	20.09^{a}±2.0	29.45^{abc}±2.4	30.45^{a}±1.8	13.90^{bcde}±1.5
Goat manure, I rate	5.73 ^{ab}±0.4	19.07^{a}±4.0	27.64^{ab}±1.8	33.58^{a}±1.3	13.98^{bcde}±1.3
Poultry manure, I rate	6.56 ^{ab}±0.3	21.33^{a}±2.9	29.15^{abc}±1.0	30.11^{a}±2.7	12.85^{bcde}±1.4
Rabbit manure, I rate	6.71 ^{ab}±1.4	19.76^{a}±2.7	26.39^{a}±1.1	32.54^{a}±1.8	14.51^{cde}±1.5
Horse manure, I rate	4.06 ^{ab}±1.7	20.99^{a}±2.2	29.75^{abc}±0.2	33.48^{a}±2.9	11.71^{abcde}±0.9
Urban bottom sediment, I rate	4.93 ^{ab}±2.5	21.27^{a}±3.8	34.21^{d}±0.5	29.90^{a}±1.0	9.70^{ab}±1.9
Rural bottom sediment, I rate	5.84 ^{ab}±0.9	20.26^{a}±2.5	32.09^{cd}±1.5	30.91^{a}±1.5	10.90^{abcd}±2.4
Sewage sludge I, I rate	7.65^{b}±0.7	20.89^{a}±3.5	28.10^{abc}±1.8	28.07^{a}±2.4	15.28^{de}±0.7
Sewage sludge II, I rate	4.92 ^{ab}±1.5	22.30^{a}±3.9	31.13^{bcd}±1.5	33.94^{a}±2.1	7.70^{a}±1.0

* Means marked with the same letter did not differ significantly across the treatments ($p < 0.05$, $n = 3$) according to the Tukey test

Table S6. Results of PCA based on enzymatic and nutrient parameters across EOM types and rates; a) Eigenvalues and variance (%) explained by the first two PCA axes; b) loading components for each variable associated with the two axes

Parameter	Axis 1	Axis 2
a)		
Eigenvalues	3.49	2.34
Percentage	43.66	29.31
Cum. Percentage	43.66	72.97
b)		
AcPh	0.355	0.397
AlkPh	0.223	-0.437
Dehydrogenases	0.323	-0.379
Am-N	0.378	0.130
Ni-N	0.482	-0.005
AvP	0.455	-0.113
AvK	0.094	-0.576
pH	-0.358	-0.384
